# Regulatory roles of differentially expressed MicroRNAs in metabolic processes in negative Lens-induced myopia Guinea pigs

**DOI:** 10.1186/s12864-020-6447-x

**Published:** 2020-01-06

**Authors:** Dadong Guo, Meihua Ding, Xiaoli Song, Yuanyuan Sun, Guoping Li, Zonghong Li, Huixia Wei, Jianfeng Wu, Wenjun Jiang, Hongsheng Bi

**Affiliations:** 1 Shandong Provincial Key Laboratory of Integrated Traditional Chinese and Western Medicine for Prevention and Therapy of Ocular Diseases, Key Laboratory of Integrated Traditional Chinese and Western Medicine for Prevention and Therapy of Ocular Diseases in Universities of Shandong; Eye Institute of Shandong University of Traditional Chinese Medicine, No. 48#, Yingxiongshan Road, Jinan, 250002 China; 2 Affiliated Eye Hospital of Shandong University of Traditional Chinese Medicine, No. 48#, Yingxiongshan Road, Jinan, 250002 China; 3 Shandong University of Traditional Chinese Medicine, No. 4655#, Daxue Road, Jinan, 250355 China

**Keywords:** Negative lens-induced myopia, microRNA profiling, Guinea pig, Peroxisome proliferator-activated receptor α, Metabolic pathway

## Abstract

**Background:**

Myopia is one of the most common vision defects worldwide. microRNAs can regulate the target gene expression, influencing the development of diseases.

**Results:**

To investigate the alterations of microRNA profiling in negative lens-induced myopia (NLIM) guinea pigs and to explore the regulatory role of microRNAs in the occurrence and the development of myopia, we first established the NLIM guinea pig model after induction for 2 weeks. Further, we isolated sclera to purify total messenger RNA (mRNA) in both NLIM and NLIM fellow sclera. Using next generation sequencing technique and bioinformatics analysis, we identified the differentially expressed microRNAs in NLIM guinea pigs, performed the bioinformatics annotation for the differentially expressed microRNAs, and validated the expression of differentially expressed microRNAs. As a result, we successfully established an NLIM model in guinea pigs, identified 27 differentially expressed microRNAs in NLIM guinea pig sclera, including 10 upregulated and 17 downregulated microRNAs. The KEGG annotation showed the main signaling pathways were closely associated with PPAR signaling, pyruvate and propanoate metabolisms, and TGF-beta signaling pathways.

**Conclusions:**

Our findings indicate that the development of myopia is mainly involved in the disorder of metabolic processes in NLIM guinea pigs. The PPAR signaling, pyruvate and propanoate metabolism pathways may play roles in the development of myopia.

## Background

Myopia (short-sightedness) is not only the most common refractive error in the eye, but also one of the main causes of visual impairment worldwide [1]. Other than causing blurred vision at distance, high myopia (excessive amount of myopia) exaggerates the risk of other ocular diseases including glaucoma, cataract, myopia degeneration as well as retinal detachment, and all of which could lead to irreversible visual loss [2]. The economic burden mainly caused by myopia is up to US$ 202 billion per year [3] and by 2050, 50 and 10% of the world population is estimated to possess myopia and high myopia, respectively [4]. Importantly, both children and adults can be affected by myopia, especially for school-age children. In East Asia, it is estimated that more than 80% of high school graduates are subjected to myopia, and approximately 20% of which possess high myopia [5–7]. Usually, the younger the children’s age at the beginning of myopia is, the more rapidly the situation will get worse and the likelihood of developing a sight-threatening complication of high myopia will increase [8]. Physiologically, the occurrence of myopia is closely related to the axial elongation of the eye, and high myopia is characterized by the localized ectasia of the posterior sclera accompanied by scleral thinning [9, 10].

The sclera is a dense, fibrous, and viscoelastic connective tissue that forms the size and shape of the eye. It can provide a strong framework which sustains the retina, stand the expansive force produced by intraocular pressure (IOP) and provide an approach for aqueous outflow [11, 12]. Hence, the sclera is crucial in the determination of the absolute size of the eye, playing a critical role in determining the refractive state of the eye. To date, both experimental outcomes and clinical evidence have confirmed the excessive ocular elongation related to myopia is attributed to the remodeling of extracellular matrix (ECM) for scleral shell [12]. The sclera is subjected to a series of structural alterations such as typically thinning, decrease in collagen fibril diameter and/or fiber dysregulation that are the outcome of changed metabolism, leading to excessive elongation of axis of the eye and visual impairment [13]. Moreover, modern theories of refractive development confirm that the sclera plays a critical role in control of the eye size and development of myopia. During the development of myopia, both optical power and axial length of the eye increase, making the refractive error more negative [14]. Moreover, scleral extracellular matrix degrades, and the sclera becomes thinner [13]. Therefore, the development of myopia is closely correlated with scleral remodeling. Nevertheless, which factors regulate this process is still unknown.

MicroRNAs (miRNAs) are abundant classes of noncoding RNA molecules that can negatively regulate messenger RNA (mRNA) at a posttranscriptional level so as to induce degradation of target mRNAs or to silence gene expression [15, 16]. Currently, it is demonstrated that miRNAs can also activate gene expression [17]. Thus, recognition of differentially expressed miRNAs will facilitate the understanding of disease progression, uncovering the pathogenic role of miRNAs. Previous studies have revealed that some diseases are closely related to the abnormal expression and regulation of miRNA-targeted mRNAs [18–20]. It is reported that some miRNAs show differential expression in the sclera of rapidly growing fetal eyes at different ages, and thus playing a fundamental role in regulating ocular growth [21]. Retinoic acid could also up-regulate miR-328 expression by regulating PAX6 gene level to affect retinal pigment epithelial and scleral cell proliferation in ocular growth, and thus influencing the myopia development [22]. So far, the detailed mechanism of myopia development involved in miRNA regulation is still unclear.

In order to understand the underlying mechanism of myopia development, we established a negative lens-induced myopia (NLIM) guinea pig model, identified the differentially expressed miRNAs in guinea pig sclera, and performed the related bioinformatics annotation. Our findings will provide new insights into the understanding of the biological process of myopia development.

## Results

### Alterations in axial length and refraction

Various parameters associated with refractive status, such as axial length, anterior chamber depth, vitreous length and crystalline lens thickness, were determined before and after myopia induction. As listed in Table 1, the results indicated that there was no significant difference in anterior chamber depth, crystalline lens thickness and vitreous length among all groups between normal control guinea pigs and NLIM subjects before induction of myopia (all *P* > 0.05, one-way ANOVA). However, after myopia induction for 2 weeks, we observed that the vitreous length, axial length, and refraction markedly changed. The vitreous length and axial length were markedly elongated in NLIM eyes as compared with those of fellow eyes, whereas the refraction of NLIM eyes was remarkably decreased compared to that of NLIM fellow eyes, and these alterations accompanied by significant differences between NLIM eyes and NLIM fellow eyes (a paired sample *t*-test, *P* < 0.0001).

**Table 1:** Comparison of the data of anterior chamber, crystalline lens thickness, vitreous length, axial length, and refraction before and after NLIM in guinea pigs

LIM	Eye	Anterior chamber depth (Mean ± SEM, mm)	Crystalline lens thickness (Mean ± SEM, mm)	Vitreous length (Mean ± SEM, mm)	Axial length (Mean ± SEM, mm)	Refraction (Mean ± SEM, D)
Before	Fellow	1.25 ± 0.05	3.39 ± 0.09	3.58 ± 0.17	8.23 ± 0. 02	3.15 ± 0.14
	NLIM	1.27 ± 0.04	3.41 ± 0.11	3.55 ± 0.11	8.24 ± 0.03	3.23 ± 0.22
After	Fellow	1.26 ± 0.03	3.61 ± 0.08	3.62 ± 0.14	8.49 ± 0.03	1.85 ± 0.20
	NLIM	1.28 ± 0.07	3.63 ± 0.06	3.74 ± 0.12^**^	8.65 ± 0.03^**^	−2.16 ± 0.20^**^

Note: Compared with the relative NLIM fellow, ^**^P < 0.0001. NLIM: negative lens-induced myopia

### Changes of posterior sclera in NLIM eyes

**Fig. 1:**
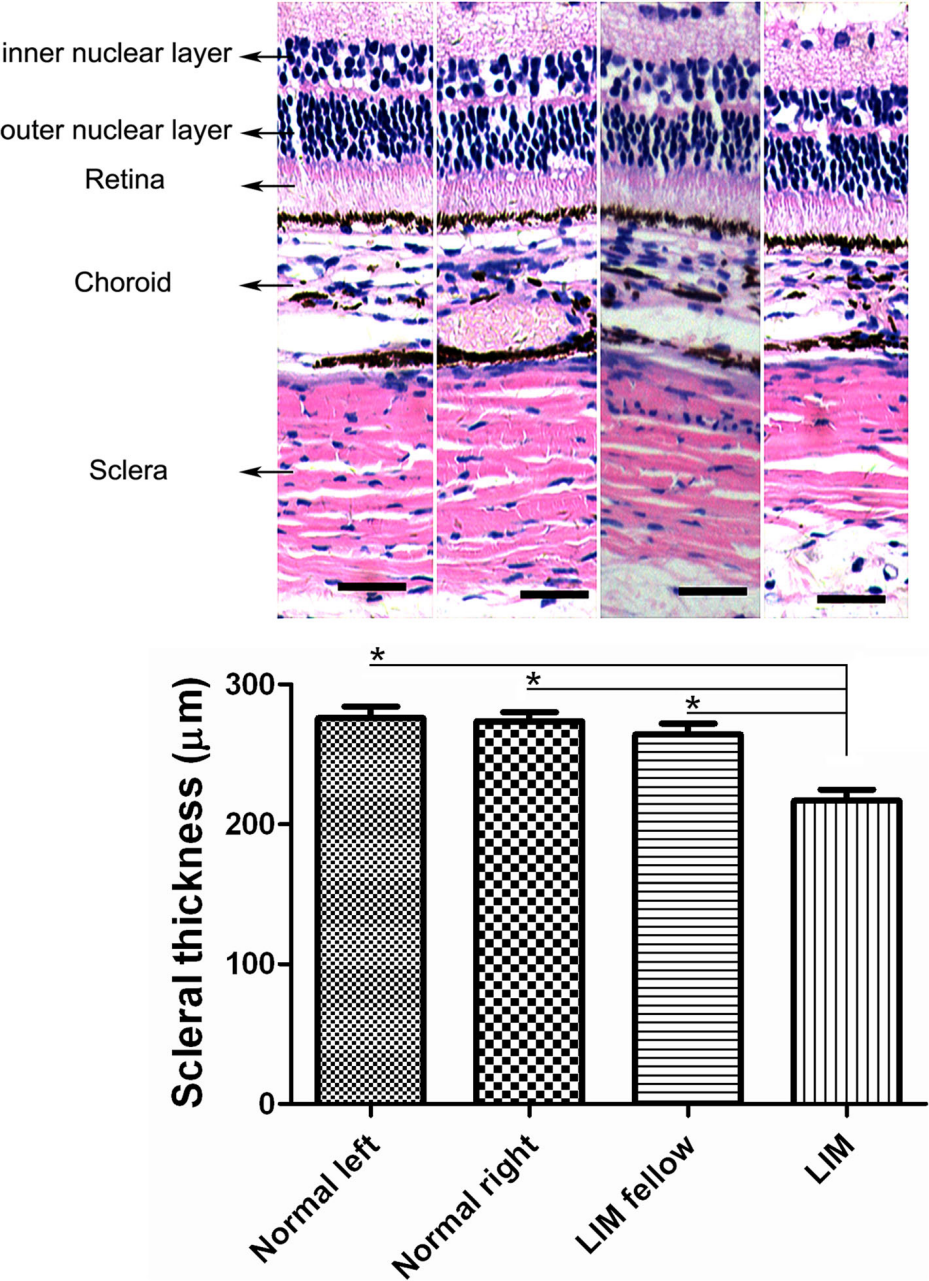
Alterations of the thickness of posterior sclera in normal control and NLIM guinea pigs. Quantitative analysis of the scleral thickness in both control and NLIM groups were performed (*n* = 6 for each group) and statistical analysis was done (lower). a = normal left eye, b = normal right eye, c = NLIM fellow eye, and d = NLIM eye. Bar = 100 μm. ^*^P < 0.01

Considering that thinned posterior sclera is an important event in the development of myopia, we further explored the alteration of the thickness of posterior sclera in NLIM guinea pigs. We observed that there was no significant alteration of the thickness in posterior sclera between normal left and right eyes (275.75 ± 8.50 μm vs. 273.50 ± 6.75 μm, *P* > 0.05; a paired sample *t*-test), and there was also no marked change between normal and NLIM fellow eyes (273.50 ± 6.75 μm vs. 267.38 ± 8.83 μm, *P* > 0.05; independent samples *t*-test). However, the sclera of NLIM eyes was significantly thinner (216.75 ± 8.17 μm) compared with those of either NLIM fellow eyes (*P* < 0.01; a paired sample *t*-test) or normal eyes (*P* < 0.01; independent samples *t*-test), and there was a statistical difference between groups (Fig. 1).

### Differentially expressed miRNA profiling

**Fig. 2:**
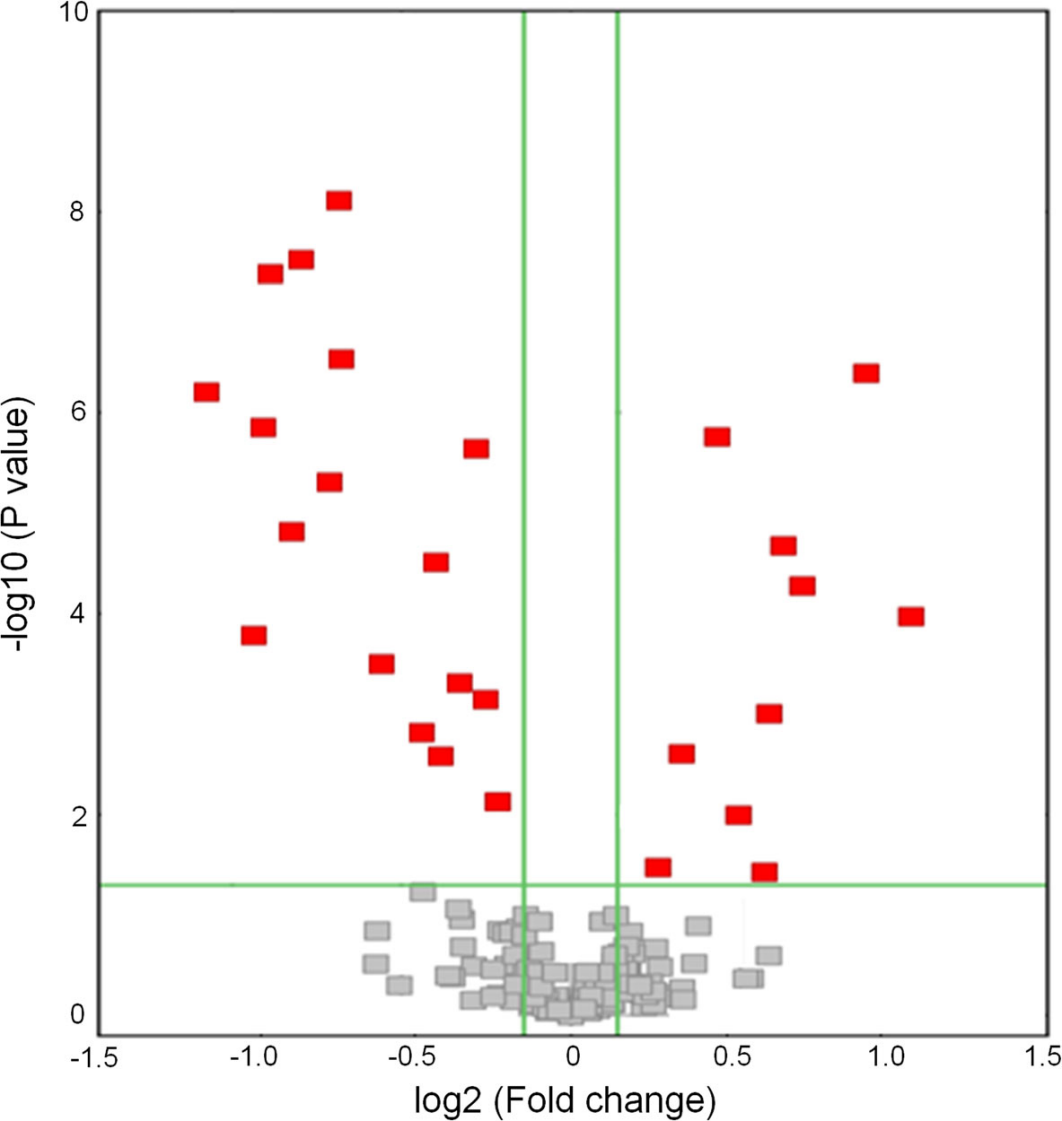
Volcano plot of differentially expressed miRNAs in NLIM sclera of guinea pigs. The red blocks are the differentially expressed miRNAs

The identification of miRNAs was performed by high-throughput sequencing data using an Illumina HiSeq 2000 platform and following prediction by algorithms. We found that 27 differentially expressed miRNAs in NLIM guinea pig sclera were significantly altered at a fold change threshold of 1.3 and FDR corrected *P*-value threshold of 0.05. The differentially expressed miRNAs were presented as a volcano plot (Fig. 2) and included 10 upregulated miRNAs and 17 downregulated miRNAs (Table 2, Additional file 2: Table S1).

**Table 2:** Differentially expressed miRNAs in negative lens-induced myopia guinea pig sclera versus fellow sclera

Mature-ID of miRNAs	Mature-sequence	Known name	Expressions
cavPor3-miR-novel-chrscaffold-107-36,268	UAACACUGUCUGGUAAAGAUG	miR-141-3p	Up
cavPor3-miR-novel-chrscaffold-111-36,350	UUGUACAUAGUAGGCUUUCAUU	miR-493-5p	Up
cavPor3-miR-novel-chrscaffold-4-5889	UUGGCUCUGCGAGGUCGGCU	miR-1842	Up
cavPor3-miR-novel-chrscaffold-7-7504	CUGCGGUGAGCCUUGAAGCCU	–	Up
cavPor3-miR-novel-chrscaffold-111-36,469	GAAUGUUGCUCGGUGAACCCCU	miR-409	Up
cavPor3-miR-novel-chrscaffold-76-32,980	CUAAGCCAGGGAUUGUGGGU	–	Up
cavPor3-miR-novel-chrscaffold-11-11,041	UUUGGCAAUGGUAGAACUCACACU	miR-182-5p	Up
cavPor3-miR-novel-chrscaffold-111-36,611	UGGAUCUUUGUCACCAGCUGAACCU	–	Up
cavPor3-miR-novel-chrscaffold-132-37,863	AAUGUACCUGGGCAAGGGUUC	miR-500-3p	Up
cavPor3-miR-novel-chrscaffold-128-37,706	UGUGCAAAUCCAUGCAAAACUG	miR-19b-3p	Up
cavPor3-miR-novel-chrscaffold-10-11,197	UAUUGCACUCGUCCCGGCCUCC	miR-92b-3p	Down
cavPor3-miR-novel-chrscaffold-111-36,353	UCCUAUAUGAUGCCUUUCCUC	rno-miR-337-3p	Down
cavPor3-miR-novel-chrscaffold-111-36,441	AAUCGUACAGGGUCAUCCACUU	miR-487b-3p	Down
cavPor3-miR-novel-chrscaffold-15-15,154	ACCGGGUGCUGUAGGCUU	–	Down
cavPor3-miR-novel-chrscaffold-12-12,421	UGGAAUGUAAGGAAGUGUGUGG	miR-206-3p	Down
cavPor3-miR-novel-chrscaffold-2-2212	GAGCAGGACGGUGGCCA	–	Down
cavPor3-miR-novel-chrscaffold-119-37,316	UGGAAUGUAAAGAAGUGUGUAU	miR-1-3p	Down
cavPor3-miR-novel-chrscaffold-111-36,472	UGGUCGACCAGUUGGAAAGU	miR-412-5p	Down
cavPor3-miR-novel-chrscaffold-68-31,730	GUGCAUGAUGACAACUG	miR-1341	Down
cavPor3-miR-novel-chrscaffold-84-33,871	UGAUUGCAUCCUCUGAGGGAGA	–	Down
cavPor3-miR-novel-chrscaffold-128-37,724	CAAAACGUGAGGCGCUGCUAU	rno-miR-322-3p	Down
cavPor3-miR-novel-chrscaffold-120-37,436	AAUGUGUAGCAGAAGACAGACU	rno-miR-511-3p	Down
cavPor3-miR-novel-chrscaffold-46-27,908	AAUGGCGCCACUAGGGUUGUGA	miR-652-3p	Down
cavPor3-miR-novel-chrscaffold-27-20,777	ACAGUAGUCUGCACAUUGGUU	miR-199a-3p	Down
cavPor3-miR-novel-chrscaffold-13-13,335	UUGGCCUACAGAAGUGACAGAC	–	Down
cavPor3-miR-novel-chrscaffold-84-33,870	CAACUCCAGGAUUCGUCGAUC	–	Down
cavPor3-miR-novel-chrscaffold-26-19,738	UUAUAAUACAACCUGAUAAGU	miR-374a-5p	Down

### Validation of differentially expressed miRNAs

**Fig. 3:**
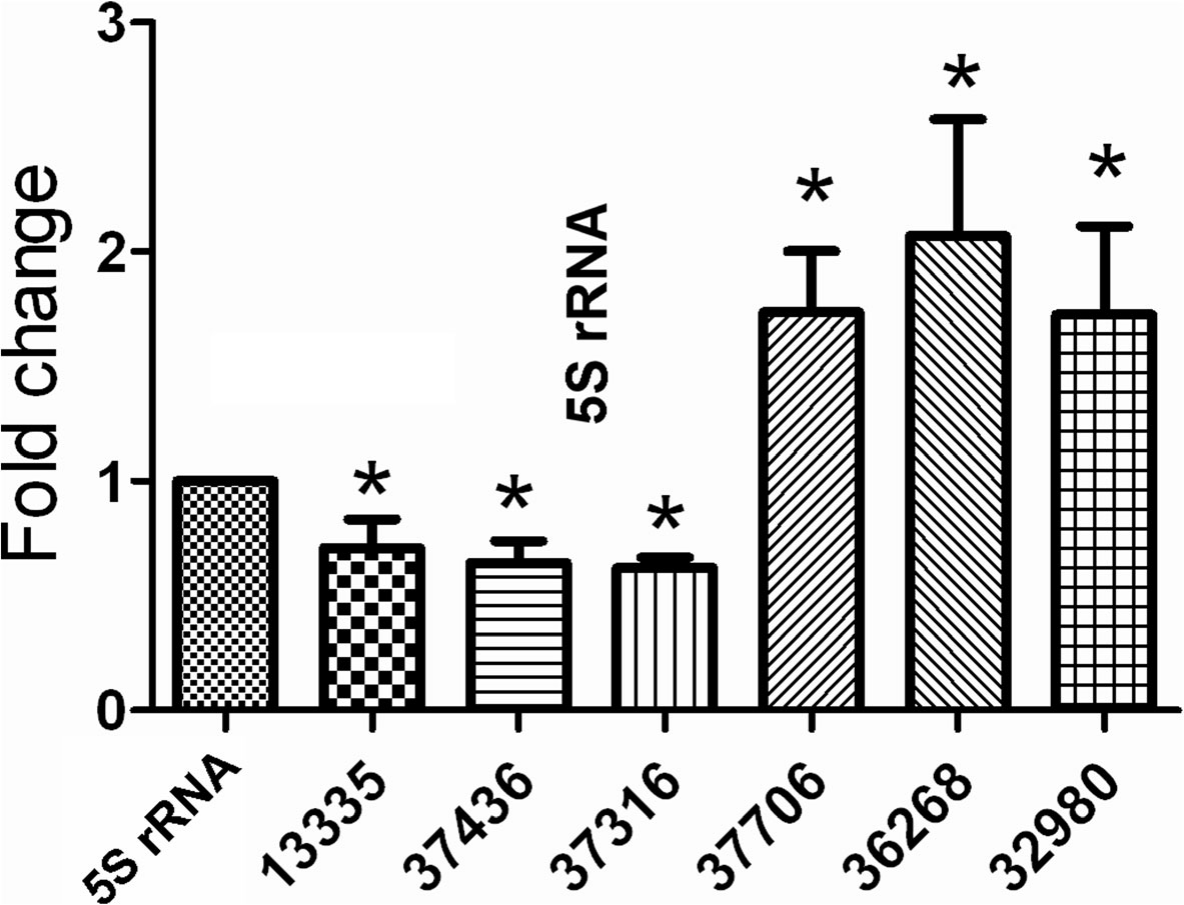
Validation of differentially expressed miRNAs in NLIM guinea pig sclera by using quantitative PCR technique. Triplicate assays were performed for each RNA sample and the relative amount of each miRNA was normalized to 5S RNA. Statistically significant difference between NLIM eyes and fellow subjects was presented by ^*^P < 0.05 (*n* = 6). 13,335 = cavPor3-miR-novel-chrscaffold-13-13,335,7436 = cavPor3-miR-novel-chrscaffold-120-37,436,37,316 = cavPor3-miR-novel-chrscaffold-119-37,316,37,706 = cavPor3-miR-novel-chrscaffold-128-37,706,36,268 = cavPor3-miR-novel-chrscaffold-107-36,268,32,980 = cavPor3-miR-novel-chrscaffold-76-32,980

Among the filtered miRNAs, 6 miRNAs were randomly selected to be validated the significantly differentially expressed in NLIM guinea pig sclera versus fellow subjects (Fig. 3), in which cavPor3-miR-novel-chrscaffold_128_37,706 (*P* < 0.01), cavPor3-miR-novel-chrscaffold_76_32,980 and cavPor3-miR-novel-chrscaffold_107_36,268 (*P* < 0.05) were upregulated miRNAs, whereas cavPor3-miR-novel-chrscaffold_119_37,316, cavPor3-miR-novel-chrscaffold_13_13,335 and cavPor3-miR-novel-chrscaffold_119_37,436 were downregulated miRNAs (*P* < 0.05), and these results were in agreement with those by sequencing and prediction by algorithms.

### GO function annotation

**Fig. 4:**
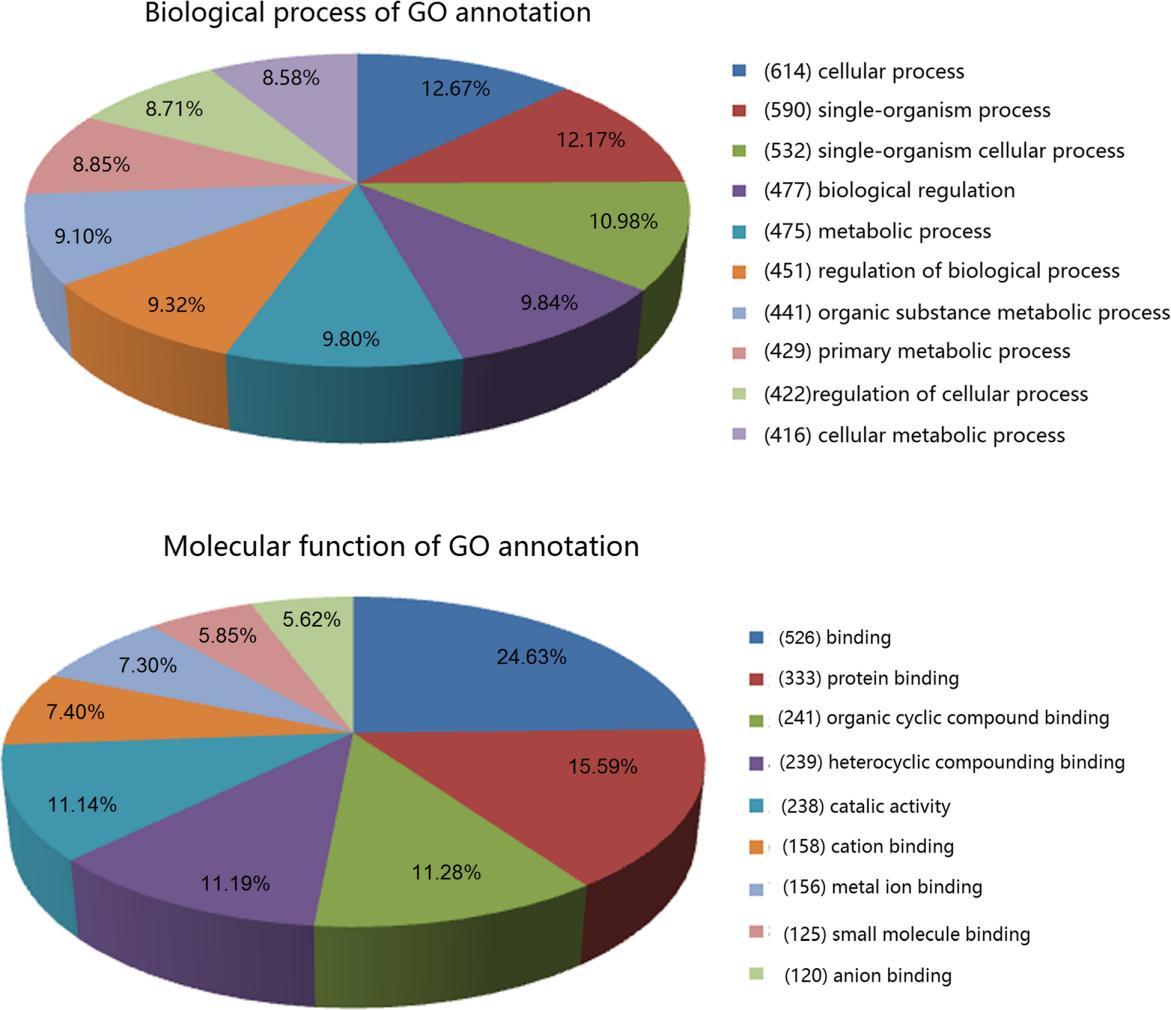
Annotations of differentially expressed microRNAs by GO annotation. Categorization of microRNA-targeted genes was performed according to the biological process and molecular function. The digit in the bracket is the number of the target genes for differentially expressed miRNAs

In the present study, the pie chart (Fig. 4) showed the top ten counts of the significant enrichment terms in molecular function (Additional file 3: Table S2) and biological process (Additional file 4: Table S3). Based on the annotation of biological process (Additional file 4: Table 3), the miRNA-targeted genes were classified into 1108 categories, including cellular process, single-organism process, single-organism cellular process, biological regulation and metabolic process, while 4847 categories were involved in binding, protein binding, and organic cyclic compounding binding.

**Table 3:** Primer sequences for differentially expressed miRNAs determined by quantitative PCR

Target gene name	Primers
5S rRNA	F: 5’TCTCGTCTGATCTCGGAAGC3’
	R: 5’GCGGTCTCCCATCCAAGTA3’
cavPor3-miR-novel-chrscaffold-13-13,335	GSP: 5’CGATTCGTTGGCCTACAGAAGTG3’
	R: 5’ATCCAGTGCAGGGTCCGAGG3’
cavPor3-miR-novel-chrscaffold-120-37,436	GSP: 5’CGCTCCGAATGTGTAGCAGAAGA3’
	R: 5’ATCCAGTGCAGGGTCCGAGG3’
cavPor3-miR-novel-chrscaffold-119-37,316	GSP: 5’GGGGGTGGAATGTAAAGAAGT3’
	R: 5’GTGCGTGTCGTGGAGTCG3’
cavPor3-miR-novel-chrscaffold-128-37,706	GSP: 5’GGGGTTGTGCAAATCCATG3’
	R: 5’GTGCGTGTCGTGGAGTCG3’
cavPor3-miR-novel-chrscaffold-107-36,268	GSP: 5’CGCCATCGTAACACTGTCTGGTA3’
	R: 5’ATCCAGTGCAGGGTCCGAGG3’
cavPor3-miR-novel-chrscaffold-76-32,980	GSP: 5’CGACATTGCCTAAGCCAGGGATT3’
	R: 5’ATCCAGTGCAGGGTCCGAGG3’

GSP is the specific primer which is matched to target gene; R is the primer that is matched to reverse transcription primer (R)

### KEGG pathway enrichment analysis

**Fig. 5:**
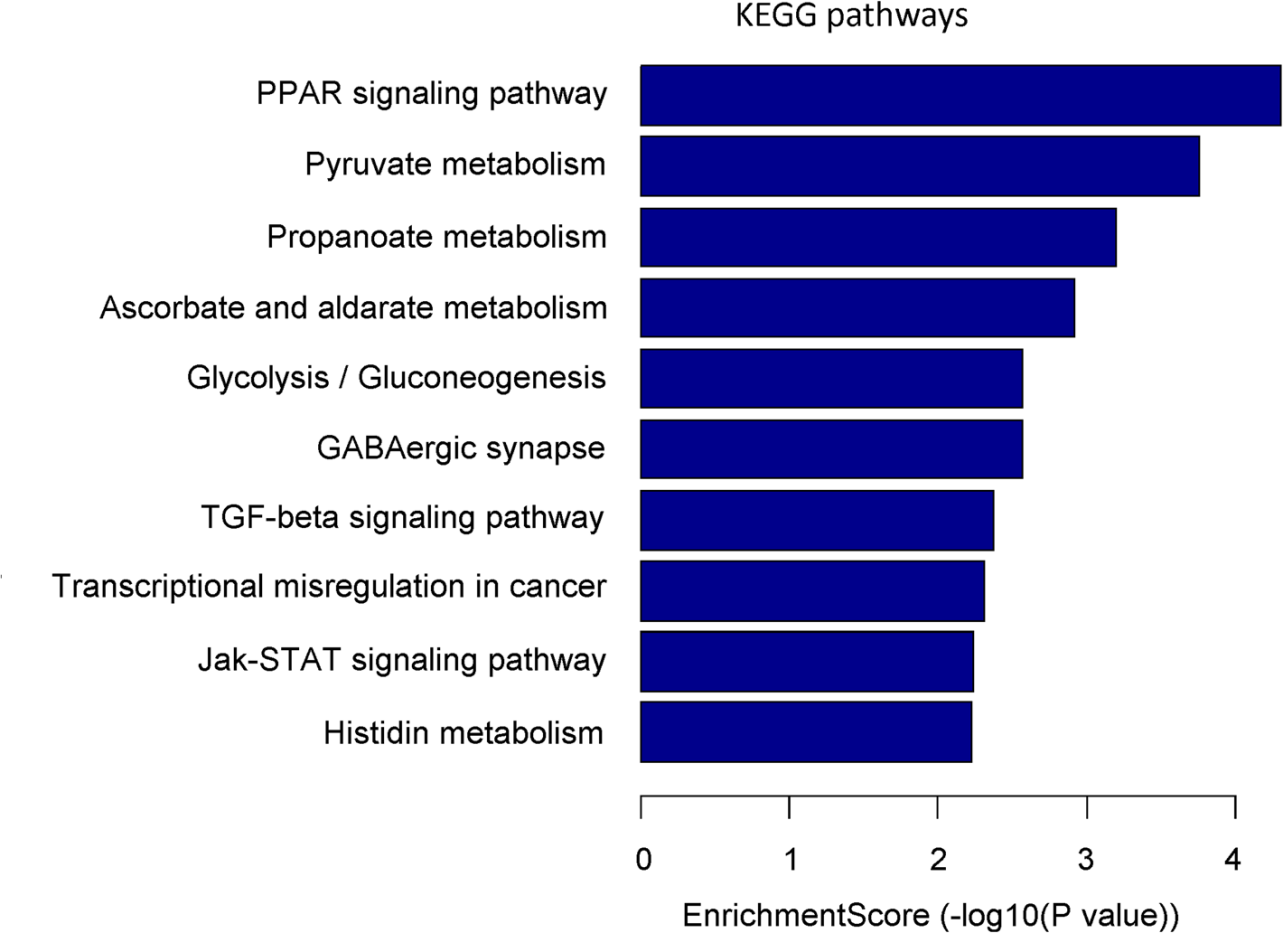
KEGG pathway enrichment analysis based on mRNAs targeted by differentially expressed miRNAs. Top ten signaling pathways are listed by the categorization

Based on the sequencing data and following prediction by algorithms, the differentially expressed miRNAs in guinea pig sclera were identified. The KEGG function annotation was performed based on the putative target genes that were regulated by miRNAs. All predicted target genes were clustered into 42 signaling pathways. Among these regulated signaling pathways, they mainly focused on the PPAR signaling, pyruvate metabolism, propanoate metabolism, ascorbate and aldarate metabolism, glycolysis/gluconeogenesis, GABAergic synapse and TGF-beta signaling pathways (Fig. 5, Additional file 5 Table 4).

**Table 4:** PPAR α primer sequences for quantitative PCR

Gene name	Primers
β-actin	F: 5′ ACCCCAAGGCCAACCGTGAGAAGATG 3’
	R: 5′ CTCGGCCGTGGTGGTGAAACTGTAGC3’
PPAR α	F: 5′ TCAAAAACCTCCGCAAACCCTTCT 3’
	R: 5′ GGCCGATCTCCGCAGCAAATGA 3’

### Expression of PPAR-α

**Fig. 6:**
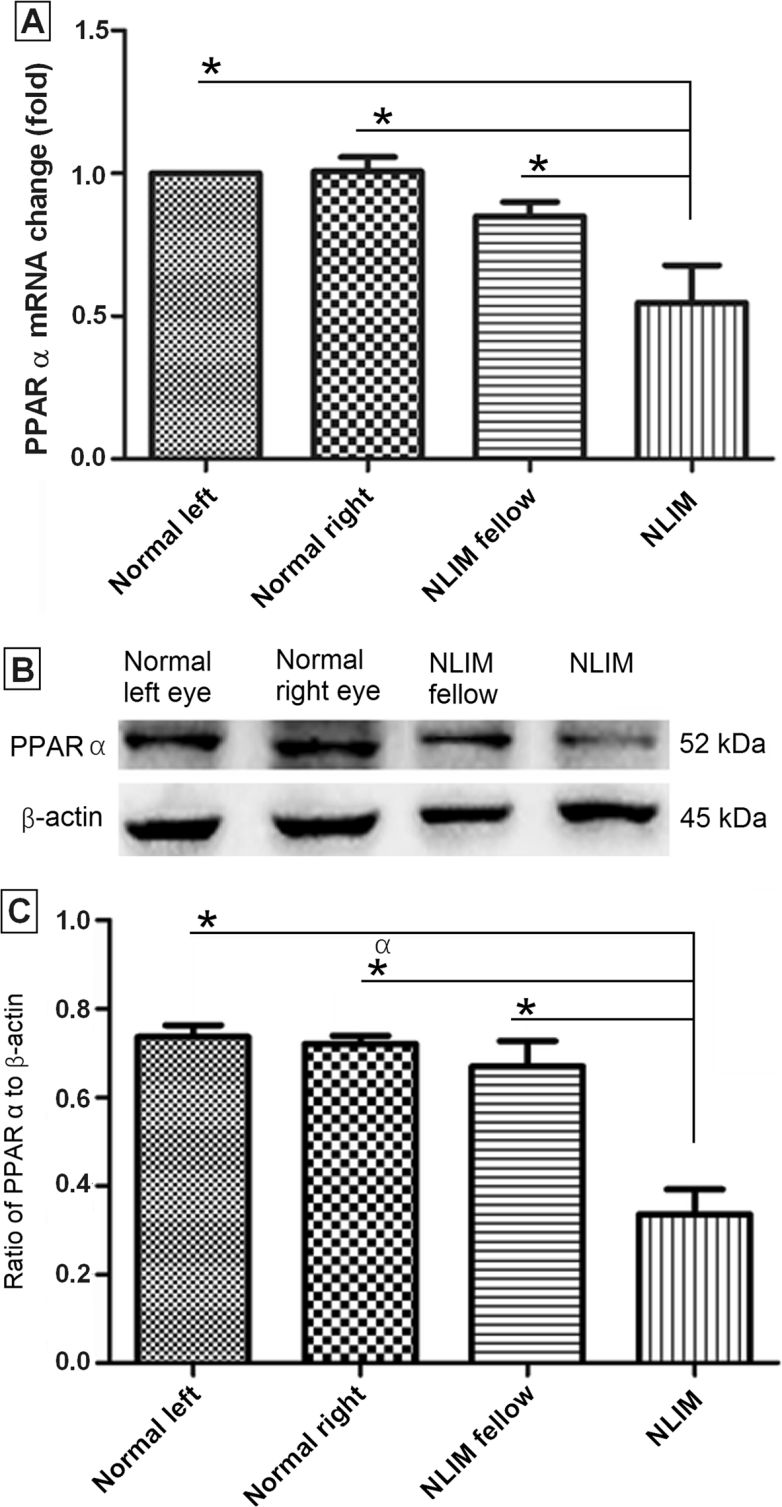
Determination of PPAR α at mRNA and protein levels in normal control and NLIM guinea pig sclera. Using pooled samples, quantitative PCR (**a**) and western blotting (**b**) analyses were done, and histogram analysis was carried out for western blotting (**c**). Data were presented as mean ± SD for mRNA and protein expressions (*n* = 6 for each group) and ^*^P < 0.05

The PPAR-α expression was further explored by using Q-PCR and western blot techniques. As shown in Fig. 6, after induction with negative lens for 2 weeks, the differences were statistically significant for PPAR-α expression between NLIM and NLIM fellow eyes. However, there was no statistically significant difference between NLIM fellow eyes and normal control eyes (Fig. 6a-c).

### Dual-luciferase reporter assay

**Fig. 7:**
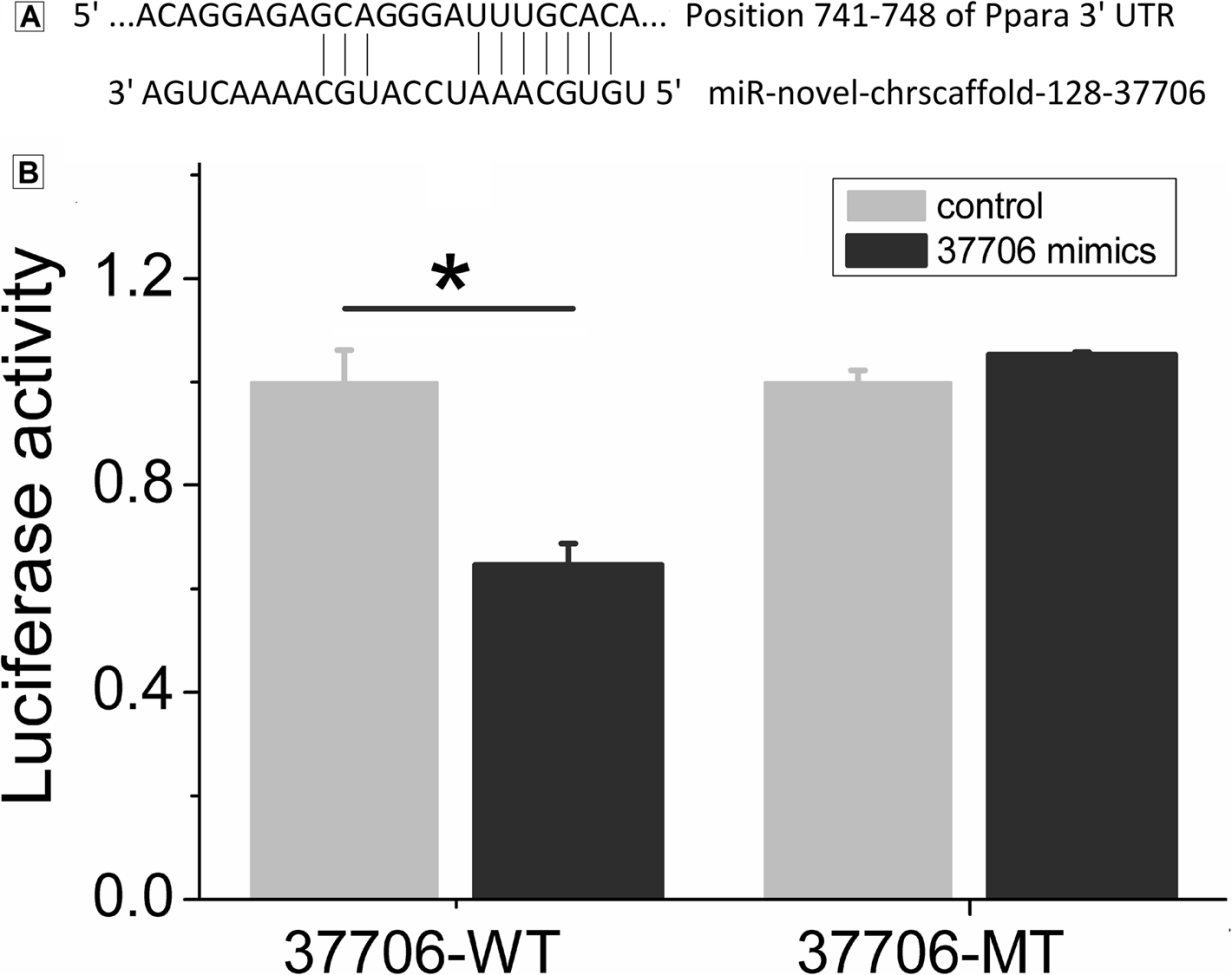
miR-novel-chrscaffold-128-37,706 targeted PPAR α. (**a**) the sequence of 3′-UTR where PPAR α mRNA bound to miR-novel-chrscaffold-128-37,706; (**b**) dual-luciferase reporter gene assay, which showed that miR-novel-chrscaffold-128-37,706 mimics could inhibit the luciferase activity of miR-novel-chrscaffold-128-37,706/PPAR α-WT plasmid. However, it had no effect on the luciferase activity of miR-novel-chrscaffold-128-37,706/PPAR α-MT. ^*^P < 0.05; WT, wild type; MT, mutant type

Using a dual-luciferase reporter assay, we further validated whether cavPor3-miR-novel-chrscaffold_128_37,706 regulates the expression of PPAR-α mRNA. The result demonstrated that the luciferase activity of the negative control sample with wild-type carriers was 1.00 ± 0.062. It was noted that the luciferase activities were 0.646 ± 0.042 and 1.00 ± 0.023 for the cavPor3-miR-novel-chrscaffold_128_37,706 group with wild-type carriers and the negative control group with mutant carriers, respectively. Regarding the cavPor3-miR-novel-chrscaffold_128_37,706 group with mutant carriers, the relative activity was 1.053 ± 0.006 (Fig. 7). These findings suggest that cavPor3-miR-novel-chrscaffold_128_37,706 (i.e., miR-19b-3p) may specifically regulate PPAR-α expression by targeting UUUGCACA at the 3′-UTR.

## Discussion

The development of myopia is a complicated process which involves the participation of many molecules and signaling pathways. Currently, some investigations of animal models have been used the tests of hypothesis as to myopia’s origins, indicating that normal growth of the eye is correlated with the roles of lower vitamin D levels, peptide factors, metabolism and accommodation [23, 24].

Studies have shown that the scleral remodeling and the consequent alteration of axial length are closely correlated with the development of myopia. The longer the axial length is, the more severe the myopia becomes [25]. To date, lens induction can efficiently disrupt the normal growth process, and induce rapid axial elongation, leading to the occurrence of myopia. It was reported that a negative lens-induced myopia model is a more credible model than that of form-deprivation myopia for the study of juvenile-onset myopia [26], and the guinea pig has been regarded as a suitable alternative mammalian model for lens-induced myopia [27]. Thus, we selected the NLIM guinea pig model to explore the underlying mechanism in the development of myopia. In this study, we have successfully established an NLIM model in guinea pigs, and noted the successful NLIM model accompanied by apparent elongated axial length, vitreous length and reduced refraction (Table 1). We also found that there is change in the crystalline lens thickness (Table 1). It is noted an increase from 3.39 mm to 3.61 mm in the fellow eye and 3.41 mm to 3.63 mm in the NLIM eye. Therefore, the changes in the refractive error cannot only be due to the increase of vitreous chamber depth and scleral thinning. The change in axial length also includes increased lens thickness. Meanwhile, the decreased thickness in posterior sclera also occurred in NLIM eyes (Fig. 1), indicating that the development of myopia is involved in the scleral remodeling.

MiRNAs play important roles in regulating gene expression in many biological and pathological processes such as cell proliferation, differentiation, apoptosis and stress response [28]. Hence, identification of differentially expressed miRNAs will facilitate the understanding of the development and pathogenesis of diseases. In order to further explore the role of miRNAs in the development of myopia, we investigated the alteration of miRNA profiling in NLIM guinea pig sclera. After induction of experimental myopia with negative lens for 2 weeks, we identified the differentially expressed miRNAs in NLIM sclera in guinea pig models. The results showed that there were 27 differentially expressed miRNAs in NLIM guinea pig sclera. Subsequent bioinformatics analysis of GO annotation demonstrated that the genes targeted by differentially expressed miRNAs were mainly related to cellular, single-organism, biological, metabolic and organic substance metabolic processes (Fig. 4), indicating the development and pathogenesis of experimental myopia involve the participation of multiple biological processes and molecules; that is to say, a lot of genes are closely associated with the development of pathogenesis of myopia. The developmental process of myopia may be correlated with the scleral remodeling and the regulation of axial length [29]. Similarly, 75 miRNAs with differential expression from the whole eye, retina and sclera were identified in form-deprivation induced myopia mice, and the differentially expressed miRNAs are associated with cell pluripotency maintenance, growth and development regulation, indicating that miRNAs play important roles in the developmental and regulatory roles in eye growth [30] Metlapally et al. observed the increased expression of let-7c, let-7e, mir-214, mir-98, mir-103, and mir-107 in fetal sclera [21], and differing in our investigations. This difference may be due to the different species, and the differentially expressed miRNAs in fetal sclera is congenital, whereas our results were obtained from the NLIM model. Moreover, using microarray technology, Tkatchenko and colleagues investigated the myopia-associated miRNA expression profiling in both sclera and retina from C57Bl/6 J mice with form-deprivation myopia. They noted there were 53 differentially expressed miRNAs in the retina, however, there was no difference in miRNA expression profiling in the sclera of mice. They noted that the differentially expressed miRNAs-targeted genes are mainly associated with transcription factors and/or regulatory proteins [31]. Mei and colleagues explored the differentially expressed miRNAs in form-deprived myopia in sclera of C57Bl/6 J mice. They found 8 differentially expressed miRNAs and enriched 1805 target genes. The functionally collaborative network indicated that the “regulation of transcription” was remarkably enriched. KEGG pathway analysis further revealed that “Axon guidance” and “TGF-β signaling pathway” were closely associated with the development of myopia of mice [32]. In our study, we only explored the miRNA expression profiling in sclera in NLIM guinea pigs. We noted that the genes regulated by differentially expressed miRNAs were closely associated with cellular process, PPAR signaling pathway, pyruvate metabolism, and TGF-β signaling pathway. The differences may be attributed to the different species and modeling methods. Currently, both lens-induced myopia and form deprivation have been widely applied in the investigation of the visual regulation in eye growth. Although there were similar results of excessive axial elongation and myopia, the visual stimuli are different: the NLIM provides a focal plane, and so is closed-loop, whereas form deprivation presents no visual feedback and so constitutes an open-loop system [33].

Homeodomain-interacting protein kinase 2 (HIPK2) has been identified as a conserved serine/threonine kinase. It can play a role in transcription, proliferation, cell differentiation, and apoptosis. Epithelial-mesenchymal transition (EMT), occurs during embryonic development, plays a crucial role in wound healing, organ fibrosis and tissue regeneration. It is reported that miR-141, a member of the miR-200 family, can inhibit EMT, and regulate renal fibrosis via TGF-β1/miR-141/HIPK2/EMT axis [34]. In our study, we noted that the upregulated miRNA cavPor3-miR-novel-chrscaffold-107-36,268 is rno-miR-141-3p. Considering that the fibrosis of sclera plays a role in scleral remodeling and further influence the development of myopia, we speculate that the upregulated cavPor3-miR-novel-chrscaffold-107-36,268 (i.e., rno-miR-141-3p) may regulate the development of myopia via TGF-β signaling pathway, and this result is also in agreement with that of KEGG pathway enrichment analysis (Fig. 5).

PPARs are nuclear receptors that are closely associated with the thyroid hormone and retinoid receptors, which play a role in regulating lipid and lipoprotein metabolism and glucose homeostasis, influencing cell proliferation, differentiation, apoptosis, and inflammation [35, 36]. In our study, we observed that cavPor3-miR-novel-chrscaffold-128-37,706 is miR-19b-3p, and it may be a potential direct regulator of PPAR α. In order to investigate whether cavPor3-miR-novel-chrscaffold-128-37,706 regulates the expression of PPAR α, we performed dual-luciferase reporter assay, and found that PPAR α is the target gene regulated by cavPor3-miR-novel-chrscaffold-128-37,706, indicating that cavPor3-miR-novel-chrscaffold-128-37,706 can play a role in PPAR signaling pathway, and thus influence the development of myopia.

Moreover, the result of the KEGG pathway enrichment analysis indicates that the differentially expressed miRNAs mainly involve PPAR signaling pathway, pyruvate metabolism, propanoate metabolism, ascorbate and aldarate metabolism, glycolysis/gluconeogenesis, GABAergic synapse, TGF-β signaling pathway (Fig. 5). That is to say, the myopic development may be correlated with the metabolic imbalance. To validate the predicated molecules of KEGG pathway enrichment analysis, we determined the PPAR α expression at both RNA and protein levels. Our results showed that PPAR α expression were reduced significantly in NLIM guinea pig sclera compared with that in NLIM fellow subject (Fig. 6), indicating that PPAR signaling pathway is involved in the development of myopia in NLIM animals.

Bertrand and colleagues found that in chicken with experimental myopia model, GW7647, a peroxisome proliferator-activated receptor α agonist, can lead to an upregulation of apoA-I level in a significant reduction in experimental myopia [37]. Thus, this result and our findings both demonstrate that PPAR signaling pathway may play a pivotal role in the development of myopia.

Nevertheless, potential limitations should be mentioned in the present study. First, there has been no a complete bioinformatics database so far on guinea pigs, therefore, we performed the relative analysis by reference to the rat bioinformatics database. Second, the animals used for measurement of scleral tissue thickness and those used for RNA-sequencing were different, and the phenotyping was not done in the eyes that scleral tissue was extracted for sequencing; this may result in deviation of the outcome. Third, the development of myopia is involved in whole visual pathways, in order to completely address the underlying mechanism of myopia, systematic investigations should be further carried out.

## Conclusions

The present work represents an initial study on the differentially expressed miRNA profiling of the sclera in an NLIM guinea pig model versus normal control subjects. The differentially expressed miRNAs and the prediction of their target genes provide support for further understanding the role of miRNAs in the NLIM guinea pig sclera and the biological processes in which they are involved. The NLIM guinea pig model is a good model species for various biological studies in the development and pathogenesis of myopia, and our findings indicate that the occurrence and the development of myopia is closely linked to the involvement of multiple signaling pathways including PPAR signaling, pyruvate and propanoate metabolisms, GABAergic synapse, glycolysis, TGF-β and Jak-STAT signaling pathways, indicating that the occurrence of myopia may be closely related to the disturbance of the metabolic processes. Taken together, our investigation provides a new insight into the underlying mechanism of the occurrence and development of myopia.

## Methods

### Experimental overview

**Fig. 8:**
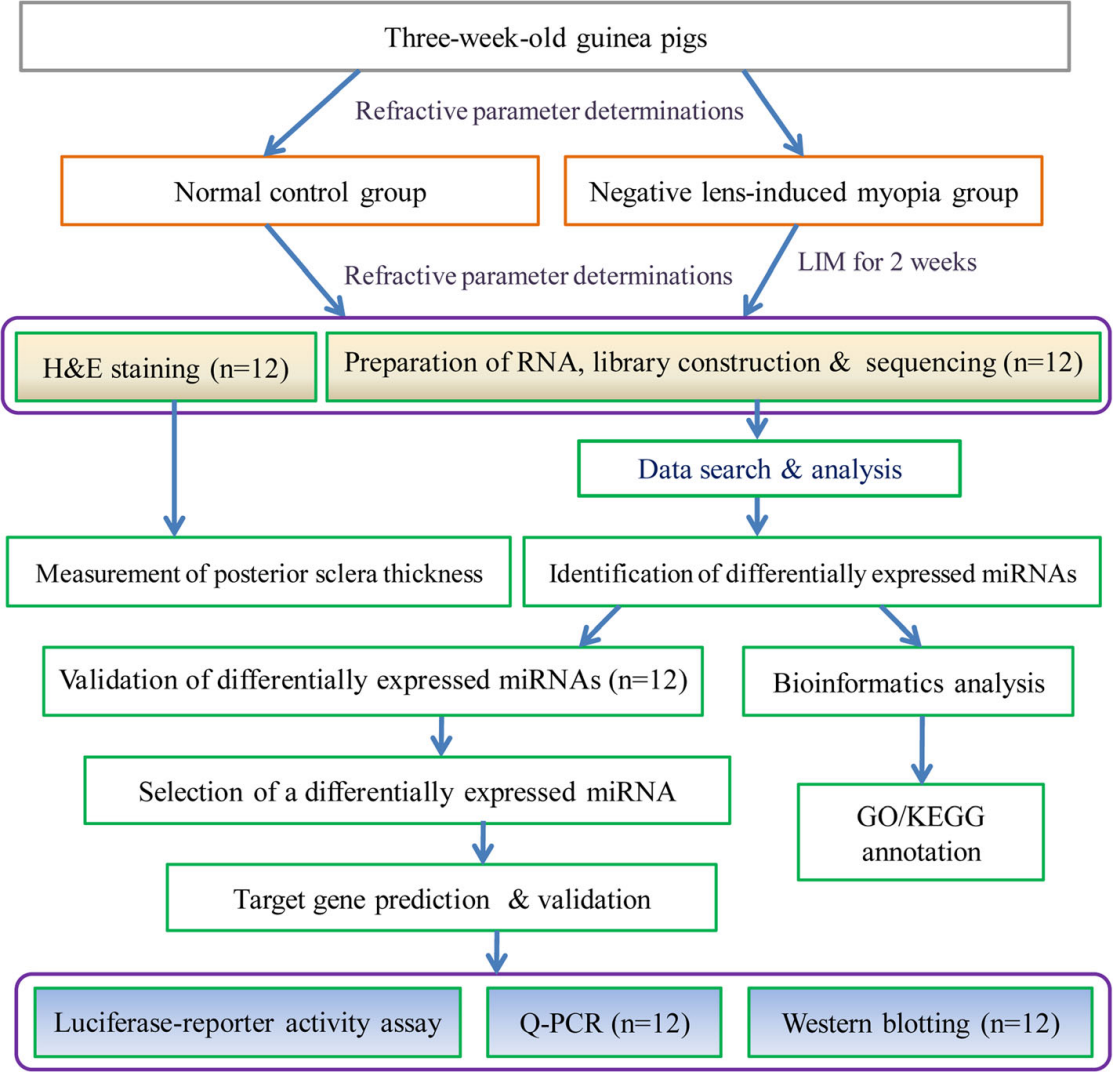
General design and workflow of the experiment. The determination at each stage includes normal control and negative lens-induced myopia (NLIM) groups, and each group contains six guinea pigs

An overview of the general experimental procedures and workflow steps was provided in Fig. 8.

### Animals

Guinea pigs (male, 180-200 g, 3-week-old) with specific pathogen-free grade were provided by Jinan Xilingjiao Laboratory Animal Co., Ltd. (Jinan, China). All guinea pigs were fed under light/dark cycles of 12 h/12 h and were allowed access to food and water freely. Experiments were approved by the Institutional Animal Care and Use Committee of Shandong University of Traditional Chinese Medicine (20150103). All guinea pigs were strictly followed by the guidelines of Care and Use of Laboratory Animals published by China National Institute of Health and the ARVO Statement for the Use of Animals in Ophthalmic and Vision Research.

### Anesthesia method

Guinea pigs were anesthetized with the injection of 3% pentobarbital (50 mg/kg) intraperitoneally until the animals were in a deep coma and pain reflex disappeared.

### Determination of refraction

A-scan ultrasonography and streak retinoscopy were applied to determine the related parameters associated with refraction status before and after induction of myopia with −10D lens. The parameters of the refraction of the eyes were obtained from the mean value of the refractive errors along the horizontal and vertical meridians of 3 repeated measurements [38, 39].

Measurements of axial length, anterior chamber depth, crystalline lens thickness and vitreous length of guinea pigs were performed by A-scan ultrasonography (Cinescan, Quantel Medical, France). Results were presented as mean value which acquired from 10 repeated measurements to minimize the error. All procedures were performed by the same professional optometrist.

### Establishment of the negative lens-induced myopia (NLIM) model

In this study, 66 guinea pigs were randomly assigned to a normal control group (NC group) and an NLIM group. Each group contained 33 guinea pigs. Before induction of myopia, the related parameters involved in refraction status were measured to exclude the congenital myopia. In the NLIM group, the right eyes for every guinea pig were covered with -10 D lens every day, while the NLIM fellow eyes were covered with plano lens. The duration of the NLIM model was maintained for 2 weeks. To ensure the effectiveness of the NLIM model in guinea pigs, all lenses were cleaned every morning and evening. At the indicted time point, the related parameters of guinea pigs in both control and NLIM groups were recorded using A-scan ultrasonography and streak retinoscopy, respectively.

### Haematoxylin and eosin (H&E) staining

After myopic induction for 2 weeks, guinea pigs in normal control and NLIM groups were euthanized and the eyes were collected (*n* = 6 for each group). After fixation in 4% paraformaldehyde in 0.1 mol/L of phosphate-buffered saline (PBS; pH = 7.2) at 4 °C for 24 h, sections were cut into 4 μm and stained with hematoxylin and eosin (H&E) solution. The observation was performed using an optical microscope (Eclipse 55i, Nikon, Japan), and the image resolution was set to 2560 × 1920 pixels. Finally, posterior scleral thickness was measured with NIS elements D 3.2 software (Nikon, Japan).

### Preparation of RNA, library construction and sequencing

For NLIM guinea pigs, sclera from both NLIM and NLIM fellow eyes in 12 guinea pigs was separately assigned to 3 mixed samples, and each mixed sample included 4 sclera samples. Firstly, NLIM guinea pigs were euthanized under anesthesia to isolate sclera. Further, sclera was ground under liquid nitrogen, followed by the purification of total RNA using TRIzol reagent (Invitrogen, Carlsbad, CA). The RNA quantity and purity were measured using a micro spectrophotometer (K5600, Beijing Kaiao Technology Development Co., Ltd., Beijing, China).

For preparation of sequencing library, ribosomal RNA was removed using the Ribo-Zero Magnetic Gold Kit (Illumina, Madison, WI, USA). Then the same total RNA was used for small RNA sequencing and total RNA of each sample was first sequentially ligated to 3′ and 5′ small RNA adapters using T4 RNA ligase. Further, using the fragmented RNA as a template, the fabrication and amplification of complementary DNA (cDNA) was done using Illumina’s proprietary RT primers and amplification primers according to the protocol of Seq-Star™ Small RNA-seq Kit (Illumina, Additional file 1). Next, the amplified fragments of about 125–145 bp were isolated and were purified on Novex 15% polyacrylamide gel electrophoresis (PAGE) gel, and then the completed libraries were ultimately quantified with an Agilent 2100 Bioanalyzer (Agilent Technologies, Palo Alto, CA).

To prepare cluster generation, every sample was diluted to a final concentration of 8 pmol/L, and then the cluster generation was done on the Illumina cBot with a TruSeq Rapid SR cluster kit (#GD-402-4001, Illumina). In the present study, high through-put sequencing was carried out on an Illumina HiSeq 2000 sequencer using the TruSeq Rapid SBS kit (#FC-402-4-2, Illumina). Raw reads were subjected to an in-house program and ACGT101-miR (LC Sciences, Houston, TX, USA) was used to remove adapter dimers, junk, low complexity, common RNA families (rRNA, tRNA, snRNA, snoRNA) and repeats. Read counts to tags per million counts (TPM) was used to normalize the expression levels of miRNAs.

### Raw data processing and predication of miRNAs

The total raw miRNA sequencing reads were first filtered using a Solexa CHASTITY quality control filter, and the Solexa CHASTITY quality filtered reads were harvested as clean reads after sequencing. The adaptor sequences were trimmed and the adaptor-trimmed-reads (> = 15 nt) were left. Further, the 3′ adapter sequence from the clean reads was trimmed, and the reads with lengths less than 15 nt were excluded. The trimmed reads in FASTA format were recorded and their length more than 15 nt was aligned to the pre-miRNA in miRBase 22.1 using Novoalign software. The obtained FASTQ sequence files were aligned to the rat reference genome in UCSC databank (RGSC6.0/rn6) using Bowtie [40]. Only unique non-duplicate reads were used for peak calling and annotation by HOMER (hypergeometric optimization of motif enrichment) software [41]. In the present study, we used miRDeep2 to predict novel miRNAs.

### Selection of differentially expressed miRNAs

All values of NLIM and normal control samples were statistically analyzed compared to those of the fellow eyes using a paired sample *t*-test. When compared the individuals of profile differences, the “fold change” and *P*-value between NLIM and fellow eyes were computed. The miRNA was excluded if the tag-count was less than 10. Those who had fold changes either > = 1.3 or < =0.76, P-value <= 0.05 were regarded as the differentially expressed miRNAs in NLIM sclera.

### Validation of differentially expressed miRNAs by quantitative PCR (Q-PCR)

In this section, another six pairs of subjects were fabricated and were used to validate the differentially expressed miRNAs. The differentially expressed miRNAs were listed in Table 2, six miRNAs including three upregulated miRNAs (i.e., cavPor3-miR-novel-chrscaffold_128_37,706, cavPor3-miR-novel-chrscaffold_76_32,980, cavPor3-miR-novel-chrscaffold_107_36,268) and three downregulated miRNAs (i.e., cavPor3-miR-novel-chrscaffold_13_13,335, cavPor3-miR-novel-chrscaffold_119_37,316, cavPor3-miR-novel-chrscaffold_120_37,436) were randomly selected to be performed Q-PCR test. 5S rRNA was as an endogenous control. Briefly, total miRNAs were collected after purification from the pooled sclera using the RNAmisi microRNA Extraction Kit (Aidlab Biotechnologies Co., Ltd., Beijing, China). cDNA synthesis was done using an Invitrogen Superscript ds-cDNA synthesis kit in accordance with the manufacturer’s instructions. The Q-PCR determination was done by a miScript SYBR-Green PCR Kit (Qiagen, Hilden, Germany). The primers were listed in Table 3. The reactions were done in a 384-well optical plate at 95 °C for 10 min, followed by a 40-cycle for 10 s at 95 °C, 60 s at 60 °C. Analysis was performed in triplicate for each sample and repeated three times. Melting curve analysis (95 °C for 10 s, 60 °C for 60 s, and 95 °C for 15 s) was applied to validate the specificity of the amplification reactions, and 5S rRNA was used as the normalized control. In the present study, the miRNA level was quantified by an ABI PRISM 7900 system (Applied Biosystems, Foster City, CA, USA), and the relevant expression level of each miRNA was acquired using a 2^-ΔΔct^ method.

### Target mRNA prediction

To further explore the potential biological function and biological processes of differentially expressed miRNAs in NLIM guinea pigs, we further predicted the potential target gene of miRNAs generated by the relevant algorithms. Considering that there is no database for guinea pig miRNAs, we selected the database related to rat as target candidates according to the literatures [42, 43]. A comprehensive strategy was employed where target miRNAs were forecasted for the differentially expressed genes by using two independent algorithms, i.e., targetscan (http://www.targetscan.org/) and miRDB (http://mirdb.org/miRDB/). The selection of predicted target mRNA was adopted using the overlapping from two algorithms mentioned above.

### Gene ontology (GO) function annotation

In accordance with the result of bioinformatics annotation, target mRNAs regulated by differentially expressed miRNAs in guinea pigs were selected. These target genes that is specific to enterology were arrowed down according to the UniGene database. Further, GO function annotation was used to organize genes into hierarchical categories and uncover the miRNA-mRNA regulatory network on the basis of the biological process and molecular function [44]. Both χ^2^ test and two-sided Fisher’s exact test allowed for the classification of the GO category. Meanwhile, false discovery rate (FDR) was used to calculate the *P*-value to correct the type I error rate. Herein, we selected a *P* value of <0.05 for both GOs and FDR.

### KEGG pathway enrichment analysis

In the present study, the predicted genes targeted to differentially expressed miRNAs were classified in accordance with Kyoto Encyclopedia of Genes and Genomes (KEGG) pathway database to annotate the possible pathways. These differentially expressed miRNA targets were collected and performed by KEGG pathway annotation (http://www.genome.jp/kegg/). In the present study, the two sided Fisher’s exact test and the χ^2^ test were employed to classify the enrichment (Re) of pathway category. The FDR was calculated to correct the *P*-value of the type I error rate. The enrichment Re was obtained using the same formula that calculated in GO analysis. We selected the pathways whose P-value and an FDR were less than 0.05. In the meantime, the regulator pathway annotation was further done based on the scoring and visualization of the pathways collected in the KEGG database (http://www.genome.jp/kegg/).

### Validation of PPAR-α by Q-PCR and western blotting

In view of the result of KEGG pathway enrichment analysis, we selected peroxisome proliferator-activated receptor (PPAR) α, predicted as a downregulated gene in guinea pig sclera under the NLIM condition and regulated by cavPor3-miR-novel-chrscaffold_128_37,706, to validate the level of both mRNA and protein. For Q-PCR analysis of PPAR α mRNA level, total RNA (*n* = 6 for each group) was extracted from both NLIM guinea pig sclera and normal control subjects using tissue/cell RNA rapid extraction kit (Sparkjade Science Co., Ltd., China). After analysis of RNA concentration and purity, the first-strand cDNA was first synthesized with 1 μg of total RNA. Further, the Q-PCR reaction was performed using LightCycler 480 SYBR Green I Master (Roche Diagnostics, IN, USA) in a 20 μl volume. The PCR reaction program was carried out by a LightCycler 480 II instrument (Roche Diagnostics GmbH, Mannheim, Germany) with an initial denaturation of 95 °C for 5 min, followed by 45 cycles of 95 °C for 20 s, 58 °C for 20 s and 72 °C for 25 s. The △△CT values of relative gene levels were calculated as fold change in mean ± standard error (SD) after normalization to respective endogenous β-actin control. The primer sequences were listed in Table 4.

Moreover, we also performed western blotting to determine the alterations of PPAR α protein before and after induction of myopia in guinea pig sclera. Briefly, pooled sclera samples (n = 6 for each group, 15 μL/lane) in both NLIM and normal control subjects were loaded onto 12% sodium dodecyl sulfate (SDS)-polyacrylamide gel electrophoresis (SDS-PAGE), and run for 90 min at 100 V, then the isolated proteins were transferred to poly (vinylidene) fluoride (PVDF) nanofiber membrane (Millipore Corporation, Bedford, Mass) at 100 V for 120 min, and then the membranes were blocked in TBST (5% nonfat milk and 0.05% Tween 20 in TBS) buffer for 1 h at room temperature, and then washed with TBST for 5 min for 5 times. Subsequently, membranes were then incubated with rabbit polyclonal antibody against PPAR α (1:400, Abcam, Cambridge, UK) overnight at 4 °C. The membranes were washed with TBST for 5 min for 5 times and then incubated with horseradish peroxidase-labeled anti-rabbit secondary antibodies (Amersham Biosciences Co., Piscataway, NJ) diluted with 5% non-fat dry milk in TBST (1:2000) at room temperature for 1 h. Next, the membrane was washed one time in TBST for 10 min followed by 2 washes for 5 min each. Finally, visualization was performed with DAB (Sigma) using the FUSION-FX7 imaging system (Vilber Lourmat, France) and quantified by the Fusion CAPT Software (Vilber Lourmat, France). Meanwhile, anti-beta actin (1:2000, Abcam, Cambridge, UK) was used as the internal loading control. The ratio of PPAR α to actin was used to standardize across samples.

Both Q-PCR and Western blotting determinations were repeated 3 times, and the values were presented as mean ± SD (standard deviation).

### Luciferase-reporter activity assay

In accordance with the manufacturer’s instructions, the products were cloned into pmiR-RB-REPORTTM vectors (Ribio Biotech, Guangzhou, China) downstream from the hRluc luciferase coding sequence. First, primers of both wild type and mutant type of PPAR α were synthesized. The primer sequences were listed as follows: forward primer of wild type: GCGGCTCGAGATTTTTCCTGAGATGGTAG, reverse primer of wild type: AATGCGGCCGCCCTGTAATTGTCTGAATCC; forward primer of mutant type: AGCAGGGAAAACGTGTGATGGCCTCCCTCCTTAC, reverse primer of mutant type: AGGCCATCACACGTTTTCCCTGCTCTCCTGTATG. The Q-PCR amplification of target gene was performed using a LightCycler 480 II system (Roche Diagnostics GmbH, Mannheim, Germany). Next, the vectors with 40 ng of 3′-UTR reporter constructs containing either wild-type or mutated binding sites and 100 nM of miR-novel-chrscaffold_128_37,706 mimic or negative control were co-transfected into 293 T cells using the Lipofectamine 2000 reagent (Invitrogen, USA). After transfection for 48 h, luciferase activity was then determined by a Dual-Luciferase Reporter Assay System Kit (Promega Biotech, Madison, WI, USA). After normalization to the internal control (hluc), the activity of hRluc was used to assess the transfection efficiency. Meanwhile, the ratios of the firefly luciferase activity to renilla activity were also calculated. For each experiment, three repeats were performed and every result was presented as mean ± SD.

### Statistical analysis

The statistical analysis was carried out using the SPSS statistical software (SPSS Version 17.0, Chicago, USA). Using a paired sample *t*-test, the statistical analysis was performed between the NLIM eyes and the fellow eyes within the same group. At the same time, statistical analysis among groups was performed using one-way ANOVA. The *P* value less than 0.05 was regarded as statistically significant.

## Supplementary information

**Additional file 1.** Seq-Star™ Small RNA-seq Kit (Illumina).

**Additional file 2: Table S1.** Comparisons of differentially expressed miRNAs between NLIM eyes and NLIM fellow eyes.

**Additional file 3: Table S2.** Result of GO bioinformatics analysis for molecular function.

**Additional file 4: Table S3.** Result of GO bioinformatics analysis for biological process.

**Additional file 5: Table S4.** Result of KEGG bioinformatics analysis for signaling pathways.

## Data Availability

All data generated or analyzed during this study are included in the current article and its supplementary information files.

## Abbreviations

BP: Biological process
CC: Cellular component
FDR: False discovery rate
GO: Gene ontology
KEGG: Kyoto Encyclopedia of Genes and Genomes
miRNAs: micrornas
mRNA: Messenger RNA
NC group: Normal control group
NLIM: Negative lens-induced myopia
PAGE: Polyacrylamide gel electrophoresis
PPAR: Peroxisome proliferator-activated receptor
PVDF: Poly (vinylidene) fluoride
Q-PCR: Quantitative PCR
SD: Standard deviation
SDS: sodium dodecyl sulfate
