# Supplementary material for: Regulatory roles of differentially expressed MicroRNAs in metabolic processes in negative Lens-induced myopia Guinea pigs
Source: BMC Genomics. 2020 Jan 6;21:13. doi: 10.1186/s12864-020-6447-x (PMC6945544; doi:10.1186/s12864-020-6447-x)
Supplement: Supplementary file 1 — Additional file 1. Seq-Star™ Small RNA-seq Kit (Illumina). [file 12864_2020_6447_MOESM1_ESM.pdf]

Arraystar Inc., 9430 Key West Avenue #128, Rockville, MD 20850, USA
Tel: 888-416-6343 • Fax: 240-314-0301• Email: info@arraystar.com • www.arraystar.com

Seq-Star™ Small RNA-seq Kit (Illumina)

Cat#: AS-MB-012 (Set A); (Set B)

Instruction Manual version 1.0

Product summary

Product description

The Seq-Star™ Small RNA-seq Kit (Illumina) contains the components and reagents, including adaptors, primers, enzymes, and buffers, for constructing small RNA sequencing libraries on Illumina sequencing platforms.

The novel library preparation procedures are optimized for small RNA biotypes less than 200 nt in length, such as miRNAs, piRNAs, tRNAs, tRNA-related fragments (tRF), and snoRNAs. The kit produces high-yield and high-quality libraries, devoid of artifacts such as adapter primer dimer formation.

Heavily modified tRNAs and tRFs will need additional RNA pretreatments to remove the RNA modifications and prepare the RNA termini before the library construction.

Starting materials

- Total RNA, 100 ng - 1 µg

Kit components

Components	24 reactions	Storage
3’ Ligation Reaction Buffer	120 µL	-20°C
3’ Ligation Enzyme Mix	12 µL	-20°C
3’ Adaptor	12 µL	-20°C
5’ Adaptor	12 µL	-20°C
5’ Ligation Reaction Buffer	12 µL	-20°C
5’ Ligation Enzyme Mix	28.8 µL	-20°C
RT Primer	12 µL	-20°C
1st Strand Buffer	96 µL	-20°C
Seq-Star™ RTase	12 µL	-20°C
RNase Inhibitor	12 µL	-20°C
2× PCR Master Mix	600 µL	-20°C
PCR Primer	28.8 µL	-20°C
Index Primer (Set A)	10 µL	-20°C
Index Primer (Set B)	10 µL	-20°C
Gel Loading Buffer (6x)	120 µL	-20°C
DNA Ladder	50 µL	-20°C
DNA Gel Elution Buffer	2.8 ml	-20°C
Linear Acrylamide	24 µL	-20°C
TE Buffer	600 µL	-20°C
Nuclease-free water	800 µL	-20°C

Additional required materials

- 3 M sodium acetate, pH5.5
- 100% ethanol
- Thermal cycler
- Agilent 2100 Bioanalyzer (optional)
- Fresh 80% ethanol
- Qiagen QIAquick® PCR Purification Kit
- Corning® Costar® Spin-X® Centrifuge Tube Filters (4.5 µm)

Library size selection options

By gel electrophoresis

- 6% TBE PAGE gel
- Nucleic acid gel stain
- Razor blade
- 0.5ml gel breaker tubes

By magnetic beads

- Arraystar Seq-Star™ DNAClean Beads (Cat# AS-MB-007), or
- Beckman Coulter Agencourt AMPure XP beads

By Sage Science Pippin Prep™

- 3% Agarose Dye Free Gel

Protocol

Part I: 3’ Adaptor Ligation

1. Prepare the following Mix in a 200µL PCR tube for each sample:

Input RNA (100 ng ~ 1 µg)	3.0 µL
3’ Adaptor	0.5 µL
**Total Volume**	**3.5 µL**

2. Mix thoroughly by gentle up and down pipetting several times.
3. Incubate at 70°C for 2 min. Transfer immediately to ice.
4. Add to the above reaction with the following mix:

3’ Ligation Reaction Buffer	5.0 µL
3’ Ligation Enzyme Mix	1.5 µL
**Total Volume**	**6.5 µL**

5. Mix thoroughly by gentle up and down pipetting several times.
6. Incubate the ligation at 25°C for 1 hour.

Part II: Reverse Transcription Primer Hybridization

7. Add the following Mix to the ligation mixture from step 6:

Nuclease-free water	2.5 µL
RT Primer	0.5 µL
**Total Volume**	**3.0 µL**

8. Run a thermal program of [75°C, 5 min; 37°C, 15 min; 25°C, 15 min] in a thermal cycler.

Part III: 5’ Adaptor Ligation

9. Aliquot 0.5 µL of 5’ adaptor into a new 200 µL PCR tube. Incubate at 70°C for 2 min to denature the adaptor.
10. Add the following Mix to the mixture from Step 8:

5’ Adaptor (denatured)	0.5 µL
5’ Ligation Reaction Buffer	0.5 µL
5’ Ligation Enzyme Mix	1.2 µL
**Total Volume**	**2.2 µL**

11. Incubate at 25°C for 1 hour.

Part IV: Reverse Transcription

12. Mix the following components in a new 200 µL PCR tube:

Adapter ligated RNA from Step 11	15.0 µL
1st Strand Buffer	4.0 µL
RNase Inhibitor	0.5 µL
Seq-Star™ RTase	0.5 µL
**Total Volume**	**20.0 µL**

13. Incubate the RT reaction at 50°C for 1 hour.
14. Proceed immediately to the next PCR amplification step.

Part V: PCR amplification

15. Add the following Mix to the RT reaction from Step 14:

2× PCR Master Mix	25.0 µL
PCR primer	1.2 µL
Index primer	1.2 µL
Nuclease-free water	2.6 µL
**Total Volume**	**30.0 µL**

16. Run a PCR thermal program:

Cycles	Temperature	Time
1	94°C	45 sec
Amplification 12~15 (see Notes)	94°C	15 sec
	62°C	30 sec
	70°C	15 sec
1	70°C	5 min
1	4°C	Hold

Notes: The number of amplification cycles should be adjusted according to the input RNA amount:

Input RNA amount	Amplification cycles
~1 µg	12
~100 ng	15

Part VI: Library Size Selection

The library is size selected for the size range characteristic for that small RNA biotype. Gel electrophoresis is precise in sizing, however it is lower in recovery and more laborious. Magnetic bead enrichment has higher recovery and is more convenient, however the sizing is less precise and may retain adaptors, primers and dimers. It is recommended to choose the

appropriate library size selection method based on the Agilent 2100 Bioanalyzer results.

Gel Electrophoresis Size Selection

1. Purify the PCR amplification product using a QIAQuick PCR Purification Kit.

2. Elute the DNA in 26.5 µL nuclease-free water.

3. Prepare a 6% TBE PAGE gel.

4. Add 5 µL 6× Gel Loading Buffer to the purified DNA and mix well.

5. Load two wells with 15 µL of the DNA for each sample.

6. Load 5 µL DNA Ladder in one well of the gel.

7. Run the gel at 120 V for 1 hour or until the blue dye reaches the bottom of the gel.

8. Stack a 0.5 ml gel breaker tube into a nuclease-free 2 mL microcentrifuge tube.

   Notes: Commercially available gel breaker tubes have several holes at the tube bottom. You can make one by puncturing 3~4 holes in the bottom of a 0.5 ml nuclease-free microcentrifuge with a sterile 21-gauge needle (Fig. 1).

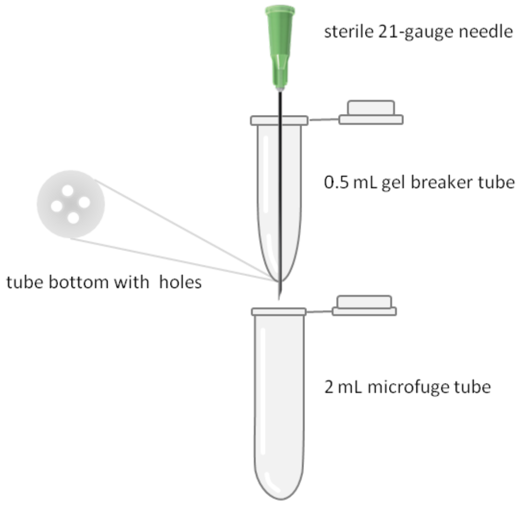

Figure 1. An assembly of stacked gel breaker tube and microcentrifuge tube.

9. Stain the gel with a nucleic acid gel stain for 2-3 min and visualize the bands on a UV transilluminator.

10. Use a razor blade to cut out the bands of interest. Place the gel slices from same sample into the 0.5 ml breaker tube assembled at Step 8.

    Notes: 140 or 150 bp adapter-ligated cDNA library bands correspond to initial 21 or 30 nt RNA fragments, respectively. For miRNAs, isolate the library at ~140 bp. For piRNAs, isolate the library at ~150 bp (Fig. 2).

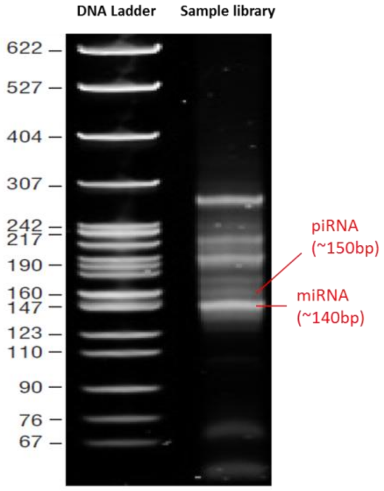

Figure 2. Gel electrophoresis of small RNA-seq library prepared from 1 µg total RNA of MCF-7 cells, showing the band sizes of miRNA and piRNA sequencing library DNAs.

11. Centrifuge the tube stack at 15,000×*g* for 3 min at room temperature to force the gel through the holes into the 2 mL tube.

    Notes: Make sure all the gel has passed through the holes.

12. Add 120 µL DNA Gel Elution Buffer to the broken-up gel in the 2ml tube.

13. Rotate in tube end-to-end orientation at room temperature for at least 2 hours or at 4°C overnight.

14. Transfer the eluate and gel debris to the top of a Spin-X column.

15. Centrifuge the filter for 2 min at > 13,000×*g*.

16. Recover eluate and add 1 µL Linear Acrylamide, 12 µL 3M sodium acetate (pH5.5), and 375 µL 100% ethanol.

17. Mix thoroughly and then allow to precipitate at -80°C for at least 30 min.

18. Spin in a microcentrifuge at > 14,000×*g* for 10 min at 4°C.

19. Remove the supernatant and wash the DNA pellet with 500 µL 80% ethanol.

20. Spin again at > 14,000×*g* for 10 min at 4°C.

21. Remove the ethanol supernatant. Air dry the pellet and resuspend in 20 µL TE buffer.

22. Check the size distribution, purity and concentration of the library with an Agilent 2100 Bioanalyzer.

Magnetic Bead Size Selection

1. Purify the library from PCR amplification using a QIAQuick PCR Purification Kit.
2. Elute the library DNA in 31.5 µL nuclease-free water.
3. To the purified 30 µL PCR reaction, add 39 µL (1.3×) of resuspended Seq-Star™ DNAClean Beads.
4. Mix thoroughly by pipetting up and down 10 times and incubate at room temperature for 5min.
5. Place the mix tube at magnetic separator until the solution becomes completely clear (1~2 minutes).
6. Carefully transfer the supernatant (69 µL) to a new nuclease-free tube. Discard the beads bound with the large DNA fragments.
7. Add 111 µL (3.7×) of resuspended DNAClean Beads to the supernatant.
8. Mix thoroughly by pipetting up and down 10 times and incubate at room temperature for 5 min.
9. Perform magnetic separation until the solution becomes completely clear (about 5 minutes). Carefully aspirate and discard the supernatant.

   Notes: The beads in this step now contain the library DNA. Do not discard!
10. Keep the tube on the magnetic separator and add 200 µL freshly prepared 80% ethanol to wash the beads. Incubate at room temperature for 30 seconds and aspirate off the supernatant.

    Caution: Do not disturb the separated magnetic beads during operation!
11. Repeat Step 10 once for a total of two washes. Make sure to remove all the remaining ethanol from the bottom of the tube.

    Optional: Air drying the beads for less than 5 minutes will ensure the removal of residual ethanol. However over drying the beads may result in dramatic loss in yields.
12. Remove tube off the magnetic separator and resuspend the beads in 21 µL TE buffer.
13. Incubate at room temperature for 2 min. Place the tube on the magnetic separator until the supernatant is completely clear from the beads.
14. Transfer the supernatant to a new nuclease-free tube.
15. Check the size, purity and concentration of the library with an Agilent 2100 Bioanalyzer.

Troubleshooting

Problem	Possible causes	Suggestion
Low library yield	Insufficient amount of starting RNA	More sensitive method should be used for measuring low sample amounts (*e.g.* Agilent Bioanalyzer).
	Contamination of enzymatic inhibitors in the RNA samples.	Ensure no residual RNA-binding inhibitors or organics in the RNA samples!
Broadly smeared PCR products on gel (Fig.2)	Degradation of input RNA	Check the RNA integrity with denaturing gel electrophoresis or Agilent 2100 Bioanalyzer.
Adapter dimers	Excessive adaptor amount	Dilute 5' and 3' adaptor before use in ligation.

Barcode index sequences (Illumina)

Set A:

1 CGATGT; #2 TGACCA; #3 ACAGTG; #4 GCCAAT; #5 CAGATC; #6 CTTGTA; #7 AGTCAA; #8 AGTTCC; #9 ATGTCA; #10 CCGTCC; #11 GTCCGC; #12 GTGAAA

Set B:

#13 ATCACG; #14 TTAGGC; #15 ACTTGA; #16 GATCAG; #17 TAGCTT; #18 GGCTAC; #19 GTGGCC; #20 GTTTCG; #21 CGTACG; #22 GAGTGG; #23 ACTGAT; #24 ATTCCTTT

Copyright ©2017 By Arraystar Inc.
