# Supplementary material for: Regulatory roles of differentially expressed MicroRNAs in metabolic processes in negative Lens-induced myopia Guinea pigs
Source: BMC Genomics. 2020 Jan 6;21:13. doi: 10.1186/s12864-020-6447-x (PMC6945544; doi:10.1186/s12864-020-6447-x)
Supplement: Supplementary file 2 — Additional file 2: Table S1. Comparisons of differentially expressed miRNAs between NLIM eyes and NLIM fellow eyes. [file 12864_2020_6447_MOESM2_ESM.docx]

Supplement Table 1 Comparisons of differentially expressed miRNAs between NLIM eyes and NLIM fellow eyes

| miRNA information | Normalized Expression Level | | Fold Change (NLIM vs NLIM fellow) | P-value | Expression |
| --- | --- | --- | --- | --- | --- |
| MATURE-ID | NLIM | NLIM fellow |  |  |  |
| cavPor3-miR-novel-chrscaffold_107_36268 | 42.33333333 | 17.66666667 | 2.396226415 | 0.033978778 | Up |
| cavPor3-miR-novel-chrscaffold_111_36350 | 352.3333333 | 242 | 1.455922865 | 0.049 | Up |
| cavPor3-miR-novel-chrscaffold_4_5889 | 33.66666667 | 17 | 1.980392157 | 0.024 | Up |
| cavPor3-miR-novel-chrscaffold_7_7504 | 20 | 14.66666667 | 1.363636364 | 0.026 | Up |
| cavPor3-miR-novel-chrscaffold_111_36469 | 285.6666667 | 190.6666667 | 1.498251748 | 0.017830971 | Up |
| cavPor3-miR-novel-chrscaffold_76_32980 | 41 | 23.66666667 | 1.732394366 | 0.046 | Up |
| cavPor3-miR-novel-chrscaffold_11_11041 | 909 | 385.6666667 | 2.356957649 | 0.01573066 | Up |
| cavPor3-miR-novel-chrscaffold_111_36611 | 54 | 33.66666667 | 1.603960396 | 0.043 | Up |
| cavPor3-miR-novel-chrscaffold_132_37863 | 36.33333333 | 13.33333333 | 2.725 | 0.021940512 | Up |
| cavPor3-miR-novel-chrscaffold_128_37706 | 334.6666667 | 192 | 1.743055556 | 0.019 | Up |
| cavPor3-miR-novel-chrscaffold_10_11197 | 15 | 23.33333333 | 0.642857143 | 0.029 | Down |
| cavPor3-miR-novel-chrscaffold_111_36353 | 17.66666667 | 29.66666667 | 0.595505618 | 0.016 | Down |
| cavPor3-miR-novel-chrscaffold_111_36441 | 34.33333333 | 48 | 0.715277778 | 0.028 | Down |
| cavPor3-miR-novel-chrscaffold_15_15154 | 874 | 1604.333333 | 0.544774569 | 0.026 | Down |
| cavPor3-miR-novel-chrscaffold_12_12421 | 39.33333333 | 138.3333333 | 0.284337349 | 0.003 | Down |
| cavPor3-miR-novel-chrscaffold_2_2212 | 25.33333333 | 46.33333333 | 0.54676259 | 0.026 | Down |
| cavPor3-miR-novel-chrscaffold_119_37316 | 228.6666667 | 582.3333333 | 0.392673154 | 0.024 | Down |
| cavPor3-miR-novel-chrscaffold_111_36472 | 21.33333333 | 40 | 0.533333333 | 0.022 | Down |
| cavPor3-miR-novel-chrscaffold_68_31730 | 15 | 26.66666667 | 0.5625 | 0.015 | Down |
| cavPor3-miR-novel-chrscaffold_84_33871 | 23 | 42.66666667 | 0.5390625 | 0.021 | Down |
| cavPor3-miR-novel-chrscaffold_128_37724 | 184.6666667 | 382.6666667 | 0.482578397 | 0.014 | Down |
| cavPor3-miR-novel-chrscaffold_120_37436 | 16 | 33 | 0.484848485 | 0.001 | Down |
| cavPor3-miR-novel-chrscaffold_46_27908 | 23.33333333 | 39.66666667 | 0.588235294 | 0.02 | Down |
| cavPor3-miR-novel-chrscaffold_27_20777 | 1070.333333 | 1779 | 0.601648866 | 0.044 | Down |
| cavPor3-miR-novel-chrscaffold_13_13335 | 19.33333333 | 30.66666667 | 0.630434783 | 0.021 | Down |
| cavPor3-miR-novel-chrscaffold_84_33870 | 28 | 54.33333333 | 0.515337423 | 0.034 | Down |
| cavPor3-miR-novel-chrscaffold_26_19738 | 22.66666667 | 45.66666667 | 0.660194175 | 0.024 | Down |
